# Integrating neutrophil-to-lymphocyte ratio into cervical cancer management: implications for prognostic assessment and therapeutic strategies – a narrative review

**DOI:** 10.1097/MS9.0000000000003963

**Published:** 2025-09-23

**Authors:** Emmanuel Ifeanyi Obeagu

**Affiliations:** Department of Biomedical and Laboratory Science, Africa University, Mutare, Zimbabwe

**Keywords:** biomarker, cervical cancer, immune response, neutrophil-to-lymphocyte ratio, prognosis

## Abstract

Cervical cancer remains a significant global health burden, particularly in low- and middle-income countries. The neutrophil-to-lymphocyte ratio (NLR), a readily accessible and cost-effective inflammatory marker, has emerged as a potential prognostic indicator in various malignancies, including cervical cancer. This narrative review aims to synthesize current evidence on the clinical relevance of NLR in cervical cancer, elucidate its underlying immunological mechanisms, and explore its implications for prognostic assessment and therapeutic decision-making. Relevant literature was identified through a comprehensive search of databases including PubMed, Scopus, and Web of Science. Emphasis was placed on studies investigating the prognostic value of NLR in cervical cancer patients, as well as those exploring the biological rationale linking inflammation and tumor progression. Elevated NLR is consistently associated with poorer survival outcomes, advanced disease stages, lymph node metastasis, and reduced treatment response in cervical cancer patients. Mechanistically, increased neutrophil counts reflect a pro-tumorigenic inflammatory state driven by cytokines such as IL-6 and VEGF, while decreased lymphocyte levels indicate compromised anti-tumor immunity. Additionally, human papillomavirus oncoproteins E6 and E7 contribute to immune evasion and may indirectly influence NLR levels. NLR holds promise as a prognostic biomarker in cervical cancer. However, significant variability in study design, cut-off values, and confounding factors limits its clinical applicability. Standardization of NLR assessment and further validation through large-scale, prospective studies are needed before routine implementation in clinical practice.

## Introduction

Cervical cancer is a significant global health issue, being the fourth most common cancer among women worldwide^[1,2]^. In 2020, there were an estimated 604 000 new cases and 342 000 deaths attributed to cervical cancer, with the highest burden observed in low- and middle-income countries[3]. The disease is primarily caused by persistent infection with high-risk types of human papillomavirus (HPV), which leads to cellular changes and, eventually, malignant transformation. Despite advances in prevention through vaccination and screening programs, cervical cancer continues to pose a substantial threat, emphasizing the need for effective biomarkers to predict disease progression and inform treatment strategies. The prognosis of cervical cancer varies widely, influenced by factors such as tumor stage, histological type, and the presence of lymph node metastasis[4]. Early-stage cervical cancer has a high cure rate with appropriate treatment, while advanced stages are associated with poorer outcomes and increased risk of recurrence[5]. Consequently, identifying reliable prognostic indicators is critical for optimizing management strategies and improving survival rates. In this context, the neutrophil-to-lymphocyte ratio (NLR) has gained attention as a potential biomarker that could aid in assessing the prognosis of cervical cancer patients. The NLR is calculated by dividing the absolute neutrophil count by the absolute lymphocyte count, providing a simple yet informative measure of the immune response[6]. An elevated NLR indicates a relative increase in neutrophils and a decrease in lymphocytes, reflecting a pro-inflammatory state that may be indicative of tumor progression. Recent studies have suggested that high NLR is associated with advanced disease stage, metastasis, and poorer survival outcomes in various malignancies, including cervical cancer. This relationship between NLR and cervical cancer prognosis warrants further investigation to determine its clinical utility.

**HIGHLIGHTS**

- **Early Prognostic Marker**: Elevated NLR serves as a cost-effective indicator of disease progression and poor prognosis in cervical cancer.
- **Treatment Stratification**: Helps tailor therapeutic approaches based on individual immune-inflammatory status.

- **Monitoring Tool**: Facilitates dynamic assessment of treatment response and cancer recurrence.
- **Immunological Insight**: Reflects the balance between tumor-promoting inflammation and anti-tumor immunity.
- **Accessible Biomarker**: Easily derived from routine blood tests, supporting widespread clinical implementation.

The mechanisms underlying the association between NLR and cervical cancer progression are complex and multifactorial[7]. Neutrophils play a crucial role in the immune response, particularly during inflammation. They can promote tumor growth and metastasis by releasing cytokines, growth factors, and reactive oxygen species that create a supportive microenvironment for cancer cells. Additionally, elevated neutrophil levels can suppress lymphocyte activity, impairing the immune system’s ability to target and eliminate tumor cells. As a result, an increased NLR may signify a compromised immune response, leading to enhanced tumor aggressiveness. Furthermore, lymphocytes, especially T cells, are essential for mediating anti-tumor immunity. A decrease in lymphocyte count, reflected by a higher NLR, suggests a diminished capacity for the immune system to recognize and combat cervical cancer cells effectively. This imbalance in the immune landscape can contribute to tumor progression and resistance to treatment. Thus, understanding the role of NLR in cervical cancer could provide insights into the immunological changes associated with the disease[8]. Several studies have examined the prognostic significance of NLR in cervical cancer, with findings indicating that elevated NLR correlates with advanced disease, lymph node metastasis, and poorer overall survival. For instance, research has shown that patients with a high NLR before treatment have a greater likelihood of experiencing treatment failure and disease recurrence. These observations suggest that NLR may serve as a valuable biomarker for risk stratification, allowing clinicians to tailor treatment approaches based on individual patient profiles^[9–11]^. Moreover, the predictive value of NLR extends beyond diagnosis and staging. Studies have demonstrated that NLR can also indicate treatment response, with decreasing NLR levels post-therapy associated with better clinical outcomes. This dynamic nature of NLR underscores its potential as a monitoring tool during the course of treatment, providing insights into the effectiveness of therapeutic interventions. Consequently, incorporating NLR into routine clinical assessments could enhance the management of cervical cancer^[10,11]^.

## Aim

The primary aim of this review is to explore the role of key biomarkers, particularly the NLR and D-dimer levels, in the prognosis and management of cervical cancer.

## Methods

This narrative review was conducted to provide an integrative overview of the prognostic and therapeutic implications of the NLR in cervical cancer. A comprehensive literature search was performed using electronic databases including PubMed, Scopus, and Web of Science. The search focused on English-language articles published up to June 2025, using key terms such as “neutrophil-to-lymphocyte ratio,” “NLR,” “cervical cancer,” “inflammation,” “prognosis,” “immune response,” and “HPV.” Peer-reviewed original articles, clinical studies, and relevant reviews were considered. Preference was given to studies that examined NLR in relation to cervical cancer prognosis, treatment response, survival outcomes, and underlying immunological mechanisms. Reference lists of selected articles were also screened to identify additional relevant publications. Given the narrative nature of this review, formal inclusion or exclusion criteria and systematic quality assessments were not applied. Instead, articles were selected based on their relevance, scientific rigor, and contribution to the conceptual understanding of NLR in the context of cervical cancer. The findings are presented thematically to highlight clinical associations, biological rationale, and future research directions.

## Limitations

While this review provides a comprehensive overview of the NLR in cervical cancer prognosis and management, several limitations must be acknowledged. First, the current literature on NLR in cervical cancer is largely composed of retrospective and single-center studies with heterogeneous patient populations, varying stages of disease, and diverse treatment protocols. This variability limits the generalizability of findings and makes it difficult to establish a universally accepted NLR cut-off value for clinical use. Additionally, many studies do not control for confounding factors such as concurrent infections, inflammatory conditions, or comorbidities, which can independently influence NLR levels. Second, the lack of standardized laboratory methods for calculating NLR and inconsistencies in the timing of measurement relative to diagnosis or treatment further complicate the interpretation of results. Moreover, while this review highlights key immunological mechanisms and clinical correlations, it is limited by its narrative nature, which inherently lacks the methodological rigor and statistical synthesis of a systematic review or meta-analysis. Consequently, selection bias may be present, and the conclusions drawn are primarily descriptive rather than quantitative. Future studies should aim to address these gaps through well-designed, prospective, and multicenter investigations that include rigorous control of confounders and standardized protocols for NLR assessment. Despite these limitations, this narrative review serves as a valuable step toward understanding the clinical relevance and potential integration of NLR into cervical cancer management.

## Neutrophil-to-lymphocyte ratio in cervical cancer

The NLR has garnered increasing attention as a potential biomarker in cervical cancer due to its simplicity and the insights it provides into the tumor microenvironment and immune status[12]. Elevated NLR indicates a predominance of neutrophils relative to lymphocytes, suggesting a systemic inflammatory response that may correlate with tumor progression and metastasis. In cervical cancer, where chronic inflammation is a recognized contributor to carcinogenesis, NLR can reflect the interplay between immune cells and tumor biology, providing valuable prognostic information. Research indicates that high NLR levels are associated with advanced disease stages in cervical cancer. For instance, studies have shown that patients with locally advanced or metastatic cervical cancer often present with significantly elevated NLR compared to those with early-stage disease. This correlation suggests that as cervical cancer progresses, the immune landscape becomes increasingly skewed towards neutrophil dominance, potentially facilitating tumor growth and dissemination. Moreover, high NLR has been linked to the presence of lymph node metastasis, reinforcing the idea that an elevated inflammatory response may promote tumor spread[13]. In addition to its prognostic implications, NLR has been studied as a predictive marker for treatment response in cervical cancer patients. Research has demonstrated that a high pre-treatment NLR can predict poorer outcomes following surgical intervention or chemoradiotherapy. For example, patients with elevated NLR at diagnosis are more likely to experience treatment failure, recurrence, or progression of disease. Monitoring NLR levels during treatment can also provide insights into therapeutic efficacy, with decreasing NLR post-treatment associated with improved survival rates. Thus, incorporating NLR into routine clinical assessments could enhance risk stratification and inform personalized treatment strategies for cervical cancer patients[14].

Neutrophils, as key players in the inflammatory response, can release pro-tumorigenic factors, such as cytokines and growth factors, which create a microenvironment conducive to tumor growth. They can also facilitate angiogenesis and metastasis by secreting matrix metalloproteinases that promote tissue remodeling[15]. Conversely, lymphocytes are crucial for mediating anti-tumor immunity, and a reduction in their levels can impair the immune system’s ability to recognize and eliminate cancer cells. This imbalance can lead to tumor progression and poorer clinical outcomes. Additionally, the role of NLR in cervical cancer may extend beyond prognosis and treatment response to encompass immunotherapy considerations[16]. As the field of cancer immunotherapy evolves, understanding the immune landscape, including the NLR, becomes critical for developing effective treatment strategies. For instance, a high NLR may indicate an immunosuppressive environment that could hinder the effectiveness of immunotherapeutic agents. Future studies should explore the potential of NLR as a predictive marker for responses to immunotherapy in cervical cancer, providing insights into patient selection and treatment planning.

## Prognostic significance of neutrophil-to-lymphocyte ratio in cervical cancer

The NLR has emerged as a significant prognostic biomarker in cervical cancer, reflecting the balance between inflammation and immune response in the tumor microenvironment[17]. Several studies have demonstrated a clear correlation between elevated NLR levels and adverse clinical outcomes in cervical cancer patients. High NLR has been associated with advanced tumor stages, increased lymph node metastasis, and a higher likelihood of recurrence, indicating that NLR can serve as an effective marker for stratifying patient risk and guiding treatment decisions[18]. Research indicates that elevated NLR may be indicative of an ongoing inflammatory response that can facilitate tumor growth and progression in cervical cancer. In particular, the presence of neutrophils in the tumor microenvironment is linked to promoting angiogenesis and enhancing tumor invasiveness, while lymphocyte depletion may signify impaired immune surveillance. Studies have shown that patients with high NLR levels exhibit poorer overall survival and progression-free survival rates compared to those with lower NLR values. This association underscores the potential of NLR as an independent prognostic factor, providing valuable insights into the disease trajectory and patient prognosis^[19,20]^. In addition to its prognostic implications, NLR can also inform treatment strategies for cervical cancer. Elevated NLR has been associated with decreased response rates to standard therapies, such as chemoradiation, suggesting that patients with high NLR may require more aggressive treatment approaches or closer monitoring during therapy. By incorporating NLR into routine clinical practice, healthcare providers can better identify high-risk patients, tailor treatment plans accordingly, and ultimately improve the overall management and outcomes of cervical cancer patients[21].

## Biological basis of NLR in cervical cancer

The NLR reflects the dynamic interplay between the pro-inflammatory and immunosuppressive arms of the immune response in the tumor microenvironment. Elevated NLR is commonly associated with poor prognostic outcomes in several malignancies, including cervical cancer, owing to its ability to mirror systemic inflammation and immune dysfunction[22]. Neutrophils play a critical role in tumor-promoting inflammation. Tumor-derived factors such as interleukin-6 (IL-6), tumor necrosis factor-alpha (TNF-α), and granulocyte colony-stimulating factor (G-CSF) stimulate neutrophil proliferation and activation. IL-6, in particular, acts as a pleiotropic cytokine that fosters tumor growth through the STAT3 signaling pathway and enhances neutrophilic infiltration. Similarly, vascular endothelial growth factor (VEGF), commonly upregulated in the hypoxic tumor milieu, contributes not only to angiogenesis but also to the recruitment of neutrophils and myeloid-derived suppressor cells (MDSCs), further amplifying the pro-tumorigenic environment[23].

MDSCs, a heterogeneous population of immature myeloid cells, are particularly enriched in tumors with elevated NLR. These cells exert potent immunosuppressive effects by inhibiting T cell proliferation and function through mechanisms involving arginase-1, inducible nitric oxide synthase (iNOS), and reactive oxygen species. By suppressing cytotoxic CD8⁺ T cells – the primary effectors of anti-tumor immunity – MDSCs contribute to immune evasion and tumor progression. Moreover, neutrophils themselves can exhibit a tumor-promoting (N2) phenotype that secretes matrix metalloproteinases and cytokines, facilitating invasion, metastasis, and remodeling of the extracellular matrix[24]. On the other hand, lymphopenia – reflected in a reduced denominator of the NLR – signals compromised adaptive immunity. A decrease in CD8^+^ cytotoxic T lymphocytes and natural killer (NK) cells impairs immune surveillance, weakening the host’s ability to target and eliminate malignant cells. This immunosuppressive state is especially relevant in cervical cancer, where persistent infection with high-risk HPV strains and expression of viral oncoproteins (e.g., E6 and E7) further disrupt antigen presentation and T cell-mediated immunity[25]. Thus, an elevated NLR can be interpreted as a composite biomarker that signifies an imbalance between tumor-promoting inflammation and tumor-suppressing immune responses.

## HPV-mediated immune modulation and NLR in cervical cancer

HPV infection is the central etiological factor in the pathogenesis of cervical cancer. Persistent infection with high-risk HPV types, particularly HPV-16 and HPV-18, leads to oncogenesis primarily through the actions of viral oncoproteins E6 and E7. These proteins not only drive cellular transformation but also profoundly alter the host immune landscape, contributing to immune evasion and tumor progression – processes that are reflected in systemic inflammatory markers such as the NLR[26]. The E6 and E7 oncoproteins interfere with antigen presentation by downregulating major histocompatibility complex class I (MHC-I) molecules, impairing cytotoxic T lymphocyte (CTL)-mediated immune surveillance. E6 promotes degradation of the tumor suppressor protein p53, while E7 inactivates retinoblastoma protein (pRb), disrupting cell cycle control. Beyond their oncogenic functions, these oncoproteins also upregulate immunosuppressive cytokines such as interleukin-10 (IL-10) and transforming growth factor-beta (TGF-β), which inhibit T cell proliferation and promote regulatory T cell (Treg) expansion[27].

In addition, HPV infection enhances the secretion of pro-inflammatory cytokines like IL-6, IL-8, and VEGF within the cervical tumor microenvironment. IL-6 and VEGF, in particular, are known to facilitate neutrophil recruitment and the expansion of MDSCs, contributing to a pro-tumorigenic milieu. This inflammatory cascade results in increased circulating neutrophils, while concurrent immunosuppressive signaling dampens lymphocyte counts – both of which elevate the NLR[28]. Moreover, chronic HPV infection contributes to immune exhaustion, with reduced numbers and functionality of CD8^+^ T cells and natural killer (NK) cells. This immune dysregulation shifts the balance away from effective tumor control, and the resultant lymphopenia reflects diminished systemic immunity[29]. Thus, HPV-driven immune modulation plays a central role in shaping both the local tumor microenvironment and systemic immune parameters. Elevated NLR in cervical cancer patients may, therefore, be partly attributed to the combined effects of viral immune evasion strategies, chronic inflammation, and suppression of adaptive immune responses. Recognizing this interplay underscores the potential of NLR not only as a prognostic biomarker but also as a reflection of HPV-mediated immunopathology in cervical cancer[30].

## Therapeutic strategies in relation to cervical cancer

Cervical cancer management has evolved significantly over the past decades, guided by disease staging, histopathological findings, and, increasingly, molecular and immunological insights. The primary goal of therapy is to achieve complete tumor eradication while preserving organ function and minimizing long-term toxicity. Treatment strategies are broadly categorized into surgical interventions, radiotherapy, chemotherapy, and, more recently, targeted and immunotherapeutic approaches. Each of these strategies plays a crucial role depending on the stage and individual characteristics of the disease[31]. For early-stage cervical cancer (stages IA to IB1), surgical intervention remains the cornerstone of treatment. Procedures such as conization, simple hysterectomy, or radical hysterectomy with pelvic lymphadenectomy are typically employed. These approaches offer high cure rates and the potential for fertility preservation in selected patients. Laparoscopic and robotic-assisted surgeries have further refined precision and recovery outcomes[21]. In locally advanced cervical cancer (stages IB2 to IVA), concurrent chemoradiation is the standard of care. External beam radiotherapy combined with intracavitary brachytherapy delivers localized tumor control, while cisplatin-based chemotherapy enhances radiosensitivity and addresses micrometastatic disease. This combined modality has significantly improved survival rates, particularly when delivered with optimal timing and dosing[32].

For metastatic or recurrent cervical cancer, systemic therapies dominate the treatment landscape. Traditionally, platinum-based chemotherapy regimens (e.g., cisplatin and paclitaxel) have been employed with modest response rates. However, the integration of targeted therapies has ushered in a new era. Bevacizumab, an anti-VEGF monoclonal antibody, has demonstrated survival benefits when added to chemotherapy, marking the first targeted agent approved for advanced cervical cancer[33]. More recently, immunotherapy has emerged as a promising frontier, particularly in patients with high PD-L1 expression or microsatellite instability. Immune checkpoint inhibitors such as pembrolizumab have shown durable responses in some cases, leading to their conditional approval. Ongoing trials are exploring combinations of immunotherapy with chemotherapy or radiotherapy to maximize synergistic effects[34]. The advent of personalized medicine is gradually transforming cervical cancer care. Molecular profiling, HPV genotyping, and inflammatory biomarkers like NLR are being explored to refine treatment decisions. Patients with specific genetic mutations or immune signatures may soon receive tailored therapies that improve outcomes while reducing toxicity[35]. Supportive care, including pain management, psychological support, nutritional guidance, and fertility counseling, is integral to comprehensive cervical cancer management. Multidisciplinary teams ensure that therapy is not only effective but also empathetic and patient-centered[36].

## Clinical implications of neutrophil-to-lymphocyte ratio in cervical cancer

The NLR has significant clinical implications in the management of cervical cancer, particularly regarding prognosis, treatment response, and personalized patient care. As a simple and cost-effective biomarker, NLR can be readily assessed through routine blood tests, making it a practical tool for clinicians. Understanding the clinical relevance of NLR can aid in enhancing patient outcomes and informing treatment strategies[37]. One of the primary clinical implications of NLR in cervical cancer is its prognostic value[38]. Studies have demonstrated that elevated NLR is associated with advanced disease stage, increased risk of metastasis, and poorer overall survival^[27,39]^. For instance, patients with high pre-treatment NLR levels tend to have more aggressive tumors and are at a greater risk for disease recurrence compared to those with lower NLR levels. This correlation underscores the importance of NLR as a prognostic indicator, allowing clinicians to stratify patients based on their risk profiles and tailor treatment approaches accordingly. In addition to prognostic implications, NLR can serve as a predictive marker for treatment response in cervical cancer. Research indicates that patients with high NLR before undergoing treatment, such as surgery or chemoradiotherapy, are more likely to experience treatment failure or disease progression. Monitoring NLR levels throughout treatment can provide insights into the effectiveness of therapeutic interventions. A decreasing NLR post-treatment may signal a favorable response, whereas persistently elevated levels could indicate treatment resistance or disease progression. Therefore, NLR can inform clinicians about the need for adjusting treatment plans based on individual patient responses^[40,41]^.

Furthermore, the integration of NLR into clinical practice can enhance the overall management of cervical cancer. By identifying patients with elevated NLR, healthcare providers can initiate closer monitoring and follow-up, ensuring timely interventions in cases of disease recurrence or progression. Additionally, NLR may help inform the decision-making process regarding the choice of therapeutic modalities. For example, patients with high NLR might benefit from more aggressive treatment strategies or adjuvant therapies to improve their chances of successful outcomes^[42,43].^ As the field of cervical cancer treatment evolves, understanding the role of NLR in the context of immunotherapy and targeted therapies is increasingly important. An elevated NLR may indicate an immunosuppressive environment that could affect the efficacy of immunotherapeutic agents. Future research should explore the potential of NLR as a predictive biomarker for responses to these emerging treatment modalities, ultimately aiding in the selection of appropriate candidates for immunotherapy based on their NLR profiles.

## Future directions

The landscape of cervical cancer research and treatment is rapidly evolving, and several future directions hold promise for improving outcomes and patient management. As we move forward, a multifaceted approach that integrates advancements in molecular biology, personalized medicine, and innovative therapeutic strategies will be essential in addressing the challenges posed by cervical cancer.

1. **Biomarker Discovery and Validation**: Future research should focus on identifying and validating additional biomarkers, including genetic, epigenetic, and proteomic signatures, that can provide insights into cervical cancer development, progression, and treatment response. Integrating biomarkers such as the NLR, D-dimer levels, and others can help create a more comprehensive profile of the disease, aiding in personalized treatment planning and improving prognostic accuracy[44].
2. **Immunotherapy Advances**: As the role of the immune system in cancer therapy gains prominence, the exploration of immunotherapeutic approaches for cervical cancer is critical. Research should focus on identifying specific immune checkpoints and developing novel immune modulators to enhance the immune response against cervical cancer cells. Clinical trials assessing the efficacy of immune checkpoint inhibitors and other immunotherapeutic agents in cervical cancer patients are essential for establishing new treatment paradigms[43].
3. **Targeted Therapies**: Future studies should investigate targeted therapies aimed at specific molecular pathways involved in cervical cancer pathogenesis. Understanding the genetic and molecular underpinnings of cervical cancer can lead to the development of personalized treatment strategies that are more effective and have fewer side effects. For instance, therapies targeting HPV-associated pathways, angiogenesis, and tumor microenvironment interactions could be explored[44].
4. **Enhanced Screening and Early Detection**: The development of more sensitive and specific screening methods is crucial for early detection and intervention in cervical cancer. Innovations in liquid biopsy techniques, which analyze circulating tumor DNA and other biomarkers, could facilitate the identification of cervical cancer at earlier stages, improving treatment outcomes. Implementing advanced screening strategies can help reduce the incidence and mortality associated with cervical cancer[36].
5. **Integration of Technology**: The use of digital health technologies, such as telemedicine, artificial intelligence (AI), and machine learning, can enhance cervical cancer management. AI algorithms can analyze large datasets from clinical and genomic studies, helping identify risk factors, predict treatment responses, and optimize patient care. Additionally, telemedicine can improve access to care, particularly for underserved populations, allowing for timely interventions and follow-up[37].
6. **Multidisciplinary Approaches**: A collaborative approach involving oncologists, gynecologists, radiologists, pathologists, and supportive care providers will be essential for comprehensive cervical cancer management. Future research should emphasize the importance of multidisciplinary care teams that address not only the biological aspects of the disease but also the psychological and social factors influencing patient outcomes[38].
7. **Health Disparities Research**: Addressing health disparities in cervical cancer is vital for ensuring equitable access to care and treatment outcomes. Future studies should focus on understanding the barriers faced by marginalized populations in accessing cervical cancer screening, diagnosis, and treatment. Implementing targeted interventions to improve health equity can help reduce the burden of cervical cancer in high-risk populations[27].
8. **Long-term Follow-up Studies**: There is a need for long-term follow-up studies to understand the late effects of cervical cancer treatments and survivorship issues. Research should explore the physical and psychological impacts of cervical cancer on survivors, including the management of treatment-related complications and the promotion of quality of life[45].

Despite growing evidence supporting the prognostic utility of the NLR in cervical cancer, its translation into clinical practice remains limited due to the absence of standardized cut-off values and inconsistent methodologies across studies. Future research should prioritize large-scale, prospective, multi-center trials that evaluate NLR in diverse populations and clinical settings. These studies are essential not only for validating NLR as a reliable prognostic biomarker but also for establishing consensus thresholds that can be integrated into clinical guidelines. Additionally, stratification of patients based on disease stage, histological subtype, and treatment modality will help clarify NLR’s prognostic significance across different clinical contexts. Standardization in both laboratory measurement and interpretation is crucial for the reproducible application of NLR in routine cervical cancer management.

## Conclusion

Cervical cancer remains a significant global health challenge, particularly in low- and middle-income countries where access to screening and treatment options is often limited. Despite advances in prevention, early detection, and treatment, the burden of cervical cancer continues to impact many lives, underscoring the urgent need for comprehensive strategies to combat this disease. The integration of innovative biomarkers, such as the NLR and D-dimer levels, offers promising avenues for improving prognosis and personalizing treatment, ultimately enhancing patient outcomes. Furthermore, ongoing research into immunotherapy and targeted therapies holds the potential to revolutionize the management of cervical cancer, providing more effective and less toxic treatment options for patients. As our understanding of the molecular mechanisms underlying cervical cancer evolves, the development of targeted interventions that address specific pathways associated with tumor progression and metastasis will be crucial. Emphasizing the importance of early detection through improved screening methods will also be vital in reducing cervical cancer incidence and mortality.

## Data Availability

Not applicable as this is a Narrative Review.

## Research registration unique identifying number (UIN)

Not applicable as this is a Narrative Review.
